# First case of hyperpigmentation in vitiligo after use of methimazole in a patient with Graves' disease^☆^

**DOI:** 10.1016/j.abd.2024.06.006

**Published:** 2025-01-27

**Authors:** Nicolau Lima, Meyer Knobel, Mayara Chinelatto, Maria Victória Quaresma

**Affiliations:** a Thyroid Unit, Division of Endocrinology and Metabolism, Faculty of Medicine, Universidade de São Paulo, Hospital das Clínicas, São Paulo, SP, Brazil; b Faculty of Medicine, Universidade de Ribeirão Preto, Guarujá, SP, Brazil; c Department of Dermatology, Hospital das Clínicas, Faculty of Medicine, Universidade de São Paulo, São Paulo, SP, Brazil

*Dear Editor,*

A 50-year-old man was seen at the endocrine outpatient clinic at Hospital das Clínicas (Universidade de São Paulo) due to hyperthyroidism in November 2020, referred by the cardiology team, which started methimazole 15 mg/day two months before. The patient had a 6-year history of generalized vitiligo, untreated for 3-years. The physical examination had no hyperthyroidism signs, thyroid enlarged on palpation, without nodules. The skin showed depigmented macules and patches involving scalp, face, neck, forearms, dorsal hand, fingers, feet, and trunk, with some hyperpigmentation in the vitiligo macules periorbital and in the scalp (Fig. 1A). Based on thyroid function tests and thyroid ultrasound (moderately hypoechoic gland without nodules, volume of 26.33 cm^3^ and increased vascularization), the diagnosis of Graves' disease was given.

**Fig. 1:**
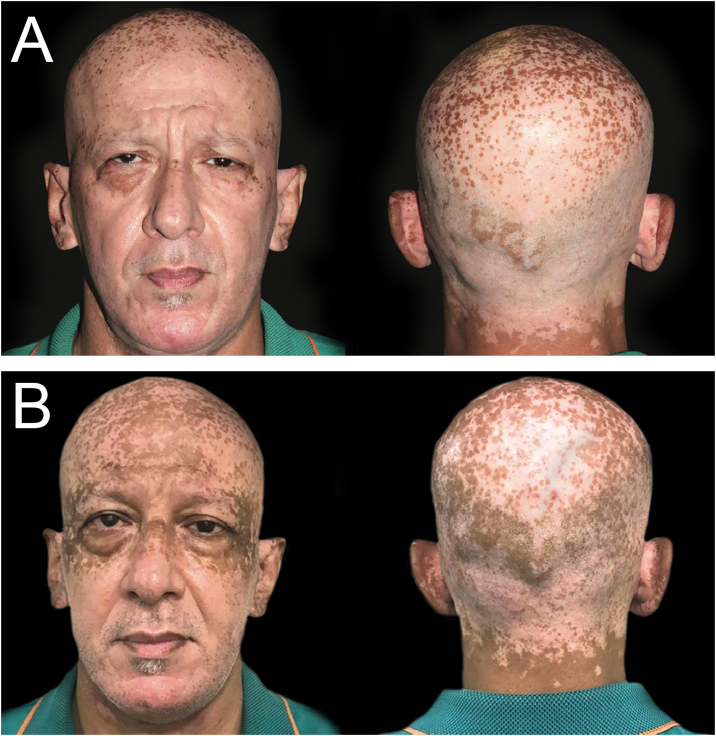
(A) Hyperpigmentation of vitiligo macules in the periorbital area and scalp, which began two months after initiation of methimazole (November 2020). (B) Five months after methimazole initiation, multiple scattered brown, well-demarcated macules and patches in sun-exposed areas in the ears, forehead, periorbital regions and scalp (February 2021).

Laboratory tests before methimazole: Thyrotropin (TSH) < 0.008 µIU/mL, serum Free T4 (FT4) 4.99 ng/mL, total Triiodothyronine (T3) 438 ng/mL, high Thyrotropin Receptor Antibody (TRAb) 3.88 IU/L, and thyroglobulin antibody undetectable.

In February 2021, while using methimazole, the patient was concerned about recent pigmentation, started two months after initiating methimazole. He denied redness, burning, itching, or other associated symptoms. On physical examination presented multiple scattered brown, well-demarcated macules, and patches in sun-exposed areas (ears, scalp, forehead, periorbital), darker than the normal surrounding skin, sparing areas covered by a face mask, used for protection against SARS-CoV-2 infection (Fig. 1B). Most of the other vitiligo lesions had some perifollicular repigmentation. Laboratory tests showed TSH < 0.02 µIU/mL, FT4 1.85 ng/mL, T3 169 ng/mL, and TRAb 2.27 IU/L.

Due to suspicion of photopigmentation an adverse event of methimazole, photoprotection was recommended. A biopsy of the brown-colored macule of the cheek was requested and showed normal stratum corneum, normal epidermis thickness, increased basal melanin pigmentation, and several melanophages in the papillary dermis (Fontana stain positive) (Fig. 2A and B). Iron staining for identification of hemosiderin deposition was negative. The use of methimazole was interrupted and was started propylthiouracil 200 mg/day.

**Fig. 2:**
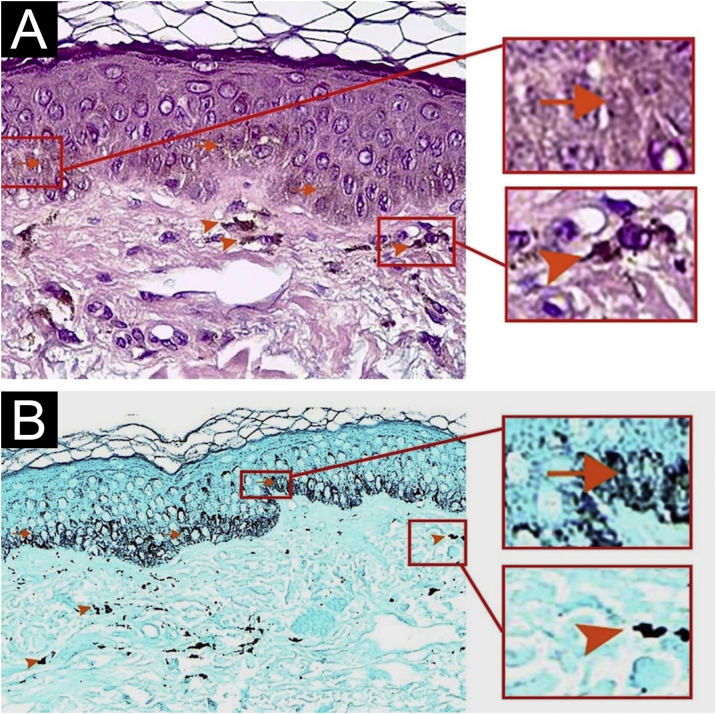
Light microscopy of a hyperpigmented patch on the cheek revealed increased melanin pigmentation of basal cells in the epidermis (arrows) and some melanophages in the superficial dermis (arrowheads). (A) Hematoxylin & eosin, 100×; (B) Fontana Masson, 100×.

In March 2021, using a hat and sunscreen was recommended (Fig. 3A). In October 2021 (TSH 0.8 µIU/mL, FT4 1.64 ng/mL), was significant lightening on the scalp, face, and ears lesions (Fig. 3B) after discontinuation of methimazole and initiated photoprotective measures.

**Fig. 3:**
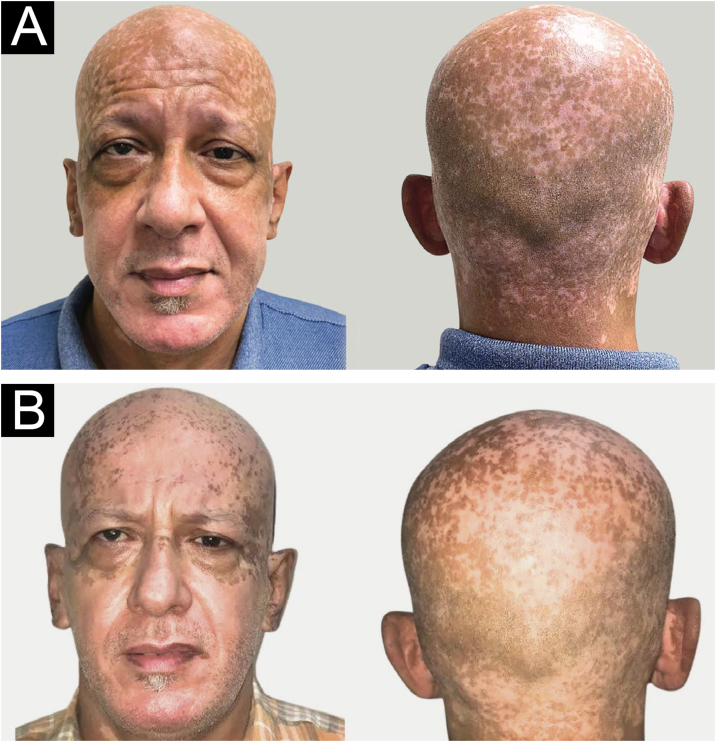
(A) Six months after starting methimazole, presence of brownish, diffuse hyperpigmentation in the entire head (March 2021). (B) Eight months after switching to propylthiouracil, in association with the use of sunscreen and hat, significant lightening of the hyperpigmented facial patches (October 2021).

Development of hyperpigmentation restricted to vitiligo areas associated with the use of methimazole has not been described in the literature as long as we know. Graves' disease is an autoimmune comorbidity associated to vitiligo, with uncommon co-occurrence.^1^ The skin hyperpigmentation, rare in Graves' disease,^2^ has been described in furosemide therapy^3^ and as an adverse effect of methimazole leaflet.

Drug-induced hyperpigmentation represents 10%–20% of acquired darkening of natural skin color.^4^ This complication is usually the consequence of hypomelanosis, increase of non-melanic origin chromophores, or endogenous and exogenous pigments deposition.^3^, ^4^ Confirming the drug involvement in skin hyperpigmentation can be challenging, especially if late-onset pigmentation or concomitant drugs use.^5^

Graves' disease hyperpigmentation may be related to pituitary ACTH increase to compensate for an accelerated manipulation of cortisol. ACTH and MSH have several amino acids in common, therefore attracting residual melanocytes in the vitiligo. ACTH increases the activation of melanocortin receptors in melanocytes. Nonetheless, epidermal melanocytes have TSH receptors sensitive to Graves' disease antibodies.^2^

The hyperpigmentation in sun-exposed skin after initiating methimazole on the case suggests drug-induced photosensitization, probably related to spontaneous pigmentation. As far as we know, the pathophysiological justification for pigmentation restricted to vitiligo areas is unknown. The methimazole substitution for propylthiouracil seems to improve pigmentation, although we cannot exclude a potential additional benefit of sunscreen and improvement of hyperthyroidism.

In drug-induced hyperpigmentation cases, the history and clinical examination should focus on the topographic distribution, histological evaluation can be considered due to patterns of melanin distribution and associated inflammatory processes, and the diagnosis can be established by resolution of the symptoms after discontinuing the drug.^4^

Hemosiderin deposition may participate in hyperthyroidism pigmentation due to increased capillary fragility in this thyroid dysfunction.^6^ In our patient, the brownish hyperpigmentation was only in sun-exposed areas of previous vitiligo patches. The marked pigmentation on the face may be related to high vellus hair density in this area, acting as a reservoir of melanocytes.

The mechanisms methimazole leads to pigmentary changes are poorly understood and deserves further investigation. Although the development of hyperpigmentation in vitiligo areas in the case suggests a drug-induced phenomenon, we cannot exclude other potential mechanisms contributing to this complication.

## Authors’ contributions

Nicolau Lima: Design and planning of the study; data collection, or analysis and interpretation of data; drafting and editing of the manuscript or critical review of important intellectual content; effective participation in research orientation.

Meyer Knobel: Drafting and editing of the manuscript or critical review of important intellectual content.

Mayara Chinelatto: Critical review of the literature.

Maria Victória Quaresma: Drafting and editing of the manuscript or critical review of important intellectual content; approval of the final version of the manuscript.

## Financial support

None declared.

## Conflicts of interest

None declared.
